# Ultra- and Moderately Hypofractionated Radiotherapy for Inoperable Cholangiocarcinoma: A Single-Institution Retrospective Analysis

**DOI:** 10.3390/curroncol32120676

**Published:** 2025-12-01

**Authors:** Saheli Saha, Cameron Lee, Zhihui Amy Liu, Michael Yan, Laura Ann Dawson, Ali Hosni Abdalaty, Jelena Lukovic, Rebecca Wong, Aisling Barry, John Kim, Jennifer J. Knox, Chaya Shwaartz, Aruz Mesci

**Affiliations:** 1 Radiation Medicine Program, Princess Margaret Cancer Centre, Toronto, ON M5G 2C4, Canada; saheli.saha@gcriindia.org (S.S.);; 2 Department of Radiation Oncology, University of Toronto, Toronto, ON M5T 1P5, Canada; 3 Medical Oncology, Princess Margaret Cancer Centre, University of Toronto, Toronto, ON M5G 2M9, Canada; 4 Toronto General Hospital, University Health Network, Toronto, ON M5G 2C4, Canada; 5 Department of Surgery, University of Toronto, Toronto, ON M5T 1P5, Canada

**Keywords:** SBRT, cholangiocarcinoma, moderately hypofractionated RT, inoperable cholangiocarcinoma

## Abstract

Systemic therapy is the mainstay of treatment in inoperable cholangiocarcinoma (CCA). The aim of this study was to evaluate the overall survival (OS), progression-free survival (PFS), recurrence patterns, and the association between biliary complications and OS in patients with inoperable, localized cholangiocarcinoma treated with radiotherapy (RT) alone. Records of patients treated between 2004 and 2022 who received a minimum of 32.5 Gy BED10 were retrospectively reviewed. Survival was estimated using the Kaplan–Meier method, and prognostic factors were assessed using univariate and multivariable analyses. A total of 56 patients (median age 67.5) were included, most of whom had intrahepatic (78.6%) CCA, and most of whom received SBRT (76.8%). The median dose was 36 Gy (BED 55 Gy), and the median OS and PFS were 20 months and 10 months, respectively. One-year local control was 92.1% and the primary site of progression was intrahepatic (64.9%). On univariate analyses, pre-radiation biliary obstruction, elevated baseline CA 19-9, larger tumor size, and age were associated with worse outcomes; on multivariable analysis, only lesion size was prognostic. Biliary complications were associated with inferior OS. These findings highlight the high intrahepatic out-of-field failure rates and suggest the incorporation of biliary-event-free survival as a clinically relevant endpoint.

## Simple Summary

Cholangiocarcinomas are a heterogeneous group of bile duct cancers. Surgery is the mainstay of treatment; however, a significant proportion of patients present with inoperable disease. Systemic therapy is the standard treatment in inoperable disease, and modern radiotherapy techniques have enabled the feasible exploration of ablative radiotherapy. In this study, we reviewed outcomes for people treated at our center with short or moderately short courses of radiotherapy. Good control of the irradiated lesions was noted at 1 year; however, recurrence in the liver but outside the treatment volume was the predominant pattern of recurrence. We also observed that overall survival was poorer in those who developed biliary problems after radiotherapy, which could be attributed to either disease progression or treatment toxicity. These findings highlight the importance of close monitoring, proactive management of biliary issues, and incorporating biliary-event-free survival as a key measure in informing care pathways.

## 1. Introduction

Cholangiocarcinomas (CCAs) are a heterogeneous group of bile duct cancers arising from the epithelial lining of intrahepatic (iCCA) (<10%), perihilar (hCCA) (50%), or distal (40%) (dCCA) biliary tracts, excluding gallbladder and ampullary cancers [1]. The incidence is 0.3–6 per 100,000 per year globally [2]. The clinical behavior of dCCAs is similar to that of gallbladder cancers, while iCCAs can sometimes mimic hepatocellular carcinoma on imaging [3]. Complete surgical resection with negative margins is the most effective treatment for CCAs [1]. In general, dCCAs and hCCAs are more likely to be resectable than iCCAs, with 63–85% of the latter being unresectable at diagnosis due to size, biliary infiltration, nodal involvement beyond pancreaticoduodenal ligament, vascular invasion, or metastases [4].

The standard management of inoperable cholangiocarcinoma is systemic therapy. A combination of gemcitabine, cisplatin, and durvalumab has been established as the standard of care, improving overall survival (OS; HR 0.8, 95% CI 0.66–0.97, *p* 0.021; 2-year OS of 25%) in iCCA in a phase 3 randomized controlled trial compared to chemotherapy alone [5]. A phase 3 randomized trial showed improved OS and resection rates with the addition of neoadjuvant chemoradiation in locally advanced gallbladder cancer (median OS 21.8 months, *p* 0.006; event-free survival 10.6 months, *p* 0.006) [6]. Modern RT techniques enable the safe delivery of focused ablative doses with stereotactic radiotherapy (SBRT) of hypofractionated RT. A recent systematic review and meta-analysis of 11 studies, both prospective and retrospective, and of small sample sizes, reported a median overall survival (OS) of 13.6 months, 1-year local control (LC) of 78.6%, with acceptable toxicities across different CCAs with SBRT [7]. NRG-GI001 attempted to assess the effect of the addition of liver-directed therapy to chemotherapy in unresectable, localized iCCAs in a randomized phase 3 study, but the study was terminated due to a lack of accrual [8].

The present study reviews our institutional practice and assesses outcomes in patients with unresectable or inoperable CCAs.

## 2. Materials and Methods

### 2.1. Inclusion/Exclusion Criteria

All patients with primary (non-recurrent), non-metastatic, localized CCA, with or without nodes, ineligible for surgical therapy, and who received moderately hypofractionated (15–20 fractions) or ultra-hypofractionated (five–six fractions) radiotherapy for local control intent were included in the analysis. Pre-treatment biopsy compatible with CCA was required for inclusion in the study.

Patients with metastatic disease, recurrent disease, mixed histology (HCC/CCA), or uncertain histology (potential metastasis from other primary causes could not be ruled out) and untreated foci (unless they were included in the treatment volume) were excluded. Patients treated solely for symptom palliation, with low doses of radiotherapy (BED10 ≤ 32.5 Gy), those who received treatment at other institutions, who received RT in the neoadjuvant/adjuvant setting or prior to liver transplantation, or those who planned for treatment with chemoradiation were excluded from analysis.

### 2.2. Data Collection

We retrospectively reviewed patients with iCCA, hCCA, or dCCA who received ultra-hypofractionated or moderately hypofractionated RT, treated at our institution between 1 January 2004 to 1 June 2022.

Data were collected from institutional electronic medical records. Characteristics of the patients including demographic (age and gender), disease (pathology, site, radiological size of the dominant lesion treated, baseline CA 19-9, and preexisting biliary obstruction) and treatment (management of obstruction, systemic therapy, and radiotherapy) details were recorded. Performance status was not clearly discernable from the charts; hence it was not collected.

At our institution, the radiotherapy simulation process involves a multiphasic contrast-enhanced CT scan and, when not contraindicated, a 3T MRI with 3 mm slice thickness. A preplanning fluoroscopy assessment is performed to evaluate respiratory motion and guide the choice of motion management strategy. Depending on the extent of motion and the patient’s ability to cooperate, one of the following is used: active breathing control (ABC), abdominal compression, or free breathing. 4DCT is routinely employed to account for tumor movement throughout the breathing cycle. The GTV is defined based on the contrast-enhancing region visible on multiphasic CT and MRI, with consideration of changes observed across different contrast phases. The CTV expands upon the GTV to include areas at risk for microscopic disease spread. A high-dose CTV is consistently delineated; the inclusion of an elective or low-dose CTV and nodal regions, when clinically indicated, is at the discretion of the treating physician. All patients were treated on a linear accelerator with 6 MV photons. Only conformal planning (IMRT and VMAT) was allowed in our standard institutional practice for any form of hypofractionated liver radiotherapy.

Patients who were lost to follow-up were censored. Recurrence patterns were noted as reported in CT scans/MRIs and recorded as local recurrence (treated site; LR), new lesions in the liver (in-liver recurrence; ILR), regional nodal recurrence (RR), and distal (extrahepatic metastasis; EHM), respectively. Recurrence or progression was defined as per RECIST criteria. In patients with disease progression in the liver, its location was classified as being within the high-dose region (95% of prescription dose), elective region (95% of elective dose where applicable), outside treated volume (10% of prescription dose or less), or marginal (between 10 and 95%) after manually registering the diagnostic scans with treatment-planning CT scans.

Biliary complications were defined as any documented biliary event (e.g., cholangitis, biliary obstruction, stent occlusion, or the need for biliary intervention) occurring after radiotherapy. Attribution to treatment vs. disease progression was not always possible and was therefore not attempted; all post-treatment events were included irrespective of their presumed etiology.

### 2.3. Statistical Analysis

The primary objectives of the study were to assess the OS and progression-free survival (PFS). The secondary objectives were to assess the percentage of LR, patterns of recurrence, post-treatment biliary toxicity, hospitalization rates, and other toxicities.

The LR, RR, ILR, and EHM were defined as the absence of disease progression at the respective sites, assessed at specified time intervals from the RT start date. The OS and PFS were calculated from the first day of RT.

The Kaplan–Meier method was used for survival analysis. Percentage recurrence rates were analyzed using the cumulative incidence function (CIF) method, with death considered a competing risk.

The Cox proportional hazard model was used for univariable analysis (UVA), with predictors including age, type of CCA (iCCA, hCCA, or dCCA), lesion size (diameter in mm), baseline and post-RT CA 19-9, pre-radiation biliary obstruction, prior chemotherapy, radiation dose, PTV volume (in cc), and overall treatment time.

Age was categorized into three groups, less than 60 years, 60–75 years, and >75 years.

Multivariable analysis was carried out using the Cox proportional hazards regression model for the factors found significant on UVA for OS and PFS.

## 3. Results

### 3.1. Pre-Radiotherapy Patient and Treatment Characteristics

**Figure 1:**
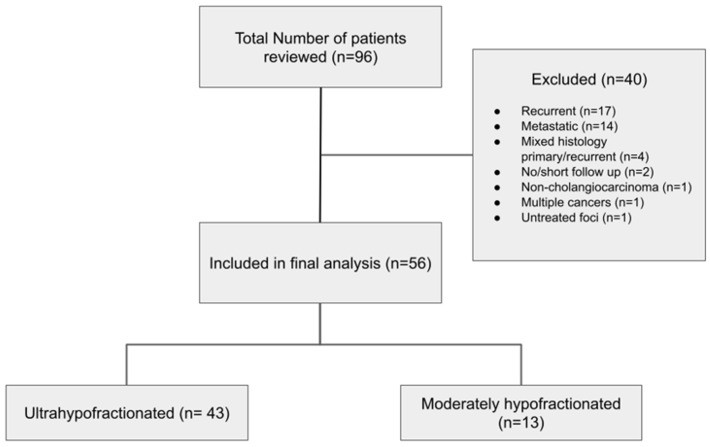
Consort diagram of the criteria for patient selection for final analysis.

A total of 96 potentially eligible patients were identified. Upon review, 40 patients were excluded (Figure 1), predominantly due to the presence of metastatic disease (*n* = 21) or prior surgical treatment (*n* = 19).

A total of 56 patients with a median age of 67.5 (range 38–90 years) were included in this analysis. The median follow-up period was 33.4 months. Baseline demographic and patient parameters are described in Table 1. The cohort had a slight male predominance (57.1%). A small proportion of the patients had a diagnosis of liver cirrhosis (*n* = eight, 14.3%), and all patients had a Child–Pugh score of A. iCCA was the most common subtype (*n* = 44; 78.6%), with eight (14.3%) and four (7.1%) patients having the diagnosis of dCCA and hCCA, respectively. The median lesion diameter was 6.3 cm (range: 8–13.9 cm), as assessed by available pre-treatment diagnostic imaging. A total of 12 patients had satellite nodules close to the primary tumor that were included in the treatment volume. The median baseline CA 19-9 was 2185 U/mL. Of the whole cohort, 17 (30.4%) patients had biliary obstruction at baseline, 8 (7.8%) underwent stenting, 6 (14.3%) had prior percutaneous drainage, and 3 (5.4%) had both procedures.

Most patients (*n* = 33; 58.9%) did not undergo systemic therapy before radiotherapy. Of these, seventeen (30.4%) were not fit due to comorbidities, age, and/or performance status. Two (3.6%) patients declined chemotherapy; the reason for not receiving chemotherapy was unknown in three (5.4%) patients. In nine (16.1%) patients, RT was planned as a first-line therapy, due to physician and/or patient preference. Thus, only twenty-three (41.1%) patients received prior chemotherapy as the initial treatment. Most patients received gemcitabine and cisplatin (GC) doublet chemotherapy (*n* = 18, 32.1%), two (3.6%) started with GC and then received durvalumab along with GC. Two (3.6%) received gemcitabine with capecitabine (gem-cap), and one patient received single-agent gemcitabine (1.8%). The median number of chemotherapy cycles was 7, ranging between 4 and 47. Only one patient (1.8%) received a second-line gem-cap following GC. Chemotherapy details are tabulated in Table 1.

The majority of patients received ultra-hypofractionated radiotherapy (SBRT—5–6 fractions; *n* = 43; 76.8%), while 13 patients (23.2%) were treated with moderately hypofractionated (mHFX) RT, ranging from 15 to 20 fractions. The median prescribed RT dose was 36 Gy (25–58.05 Gy) in 6 (5–20) fractions, with a BED10 of 55 Gy (37.5–102.6 Gy) for the whole cohort. For the SBRT cohort, the median prescribed dose was 32 Gy (25–54), while for the mHFX cohort, the median dose was 52.5 Gy (36–58.05 Gy), corresponding to a median BED10 of 51.2 Gy (37.5–102.6 Gy) and 70.9 Gy (46.8–80.5 Gy), respectively. Overall treatment times were 12 days (10–24) for SBRT and 21 days (16–22) for mHFX, respectively. Most patients completed treatment, but a small minority (*n* = five; 8.9%) did not complete RT and received a median of 40% (12 Gy, BED10 Gy 20 Gy) of their planned dose of RT. Treatment volumes were, overall, similar for the two cohorts; the median high-dose CTV volume was 165.6 cc (5.7–2320.2 cc) for the SBRT cohort and 138.9 cc (39.7–309 cc) for the mHFX cohort (*p* 0.6). The corresponding PTV volumes were 363.3 cc (14.3–3044.2 cc) for the SBRT cohort and 331 cc (69.3–677.4 cc) for the mHFX cohort (*p* 0.9). The mean (non-GTV) liver dose was 13.7 Gy (1.3–21 cc) for the SBRT group and 22.4 Gy (9.2–23.7 cc) for the mHFX cohort. Radiation details, as above, plus luminal GI doses (D0.5 cc) are described in Table 2.

### 3.2. Outcomes and Toxicity

**Figure 2:**
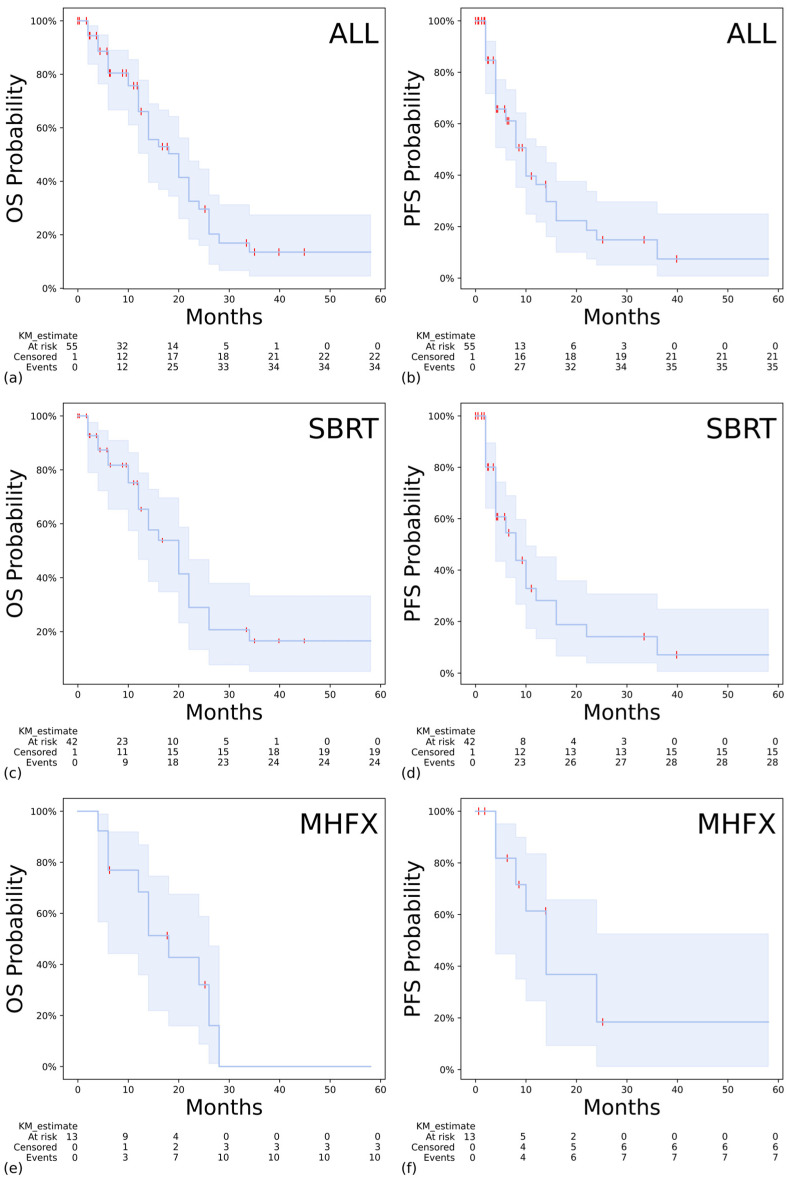
KM plots for OS and PFS. The dark blue line represents the median, and the shaded area represents the 95% CI. The red ticks denote censoring. (**a**) OS in the entire cohort, (**b**) PFS in the entire cohort, (**c**) OS in the subgroup treated with SBRT, (**d**) PFS in the subgroup treated with SBRT, (**e**) OS in the subgroup treated with mHFX, and (**f**) PFS in the subgroup treated with mHFX.

The median overall survival (mOS) was 20 months (95% CI: 14–22) and the median progression-free survival (mPFS) was 10 months (95% CI: 6–14) for the whole cohort (Figure 2a,b). The two-year OS and PFS were 29.6% and 14.9%, respectively. In the group treated with SBRT, the mOS was 20 months (95% CI: 12–22) and the mPFS was 8 months (95% CI: 4–10) (Figure 2c,d). For the mHFX cohort, the mOS and mPFS were 18 months (95% CI: 6–26) and 14 months (95% CI: 4–not estimable), respectively (Figure 2e,f).

**Figure 3:**
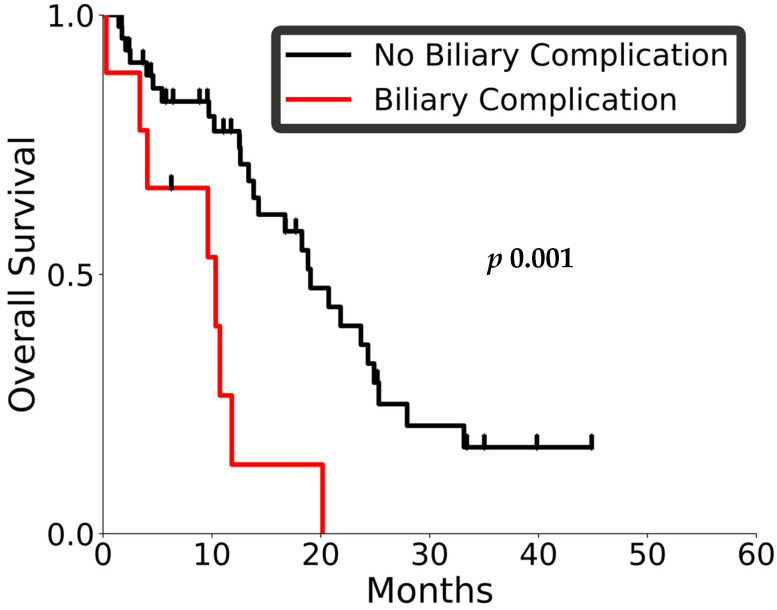
KM plots for the correlation of OS with biliary events. The black line denotes those who did not have biliary complications, and the red denotes those with biliary complications. The ticks denote censoring.

Of these patients, nine (16.1%) experienced post-radiotherapy biliary complications; five of the patients had radiologically confirmed disease progression, four did not. The median overall survival (OS) in patients with biliary complications was 10.4 months (95% CI: 0.3–11.8), compared to 19.1 months (95% CI: 13.8–24.3) in those without such events. A Kaplan–Meier analysis demonstrated a statistically significant difference in OS between the two groups (log-rank *p* 0.001; Figure 3). On Cox proportional hazards modeling, the presence of a biliary complication was associated with significantly inferior OS (HR: 3.9, 95% CI: 1.6–9.2; *p* 0.002), indicating a nearly fourfold-higher hazard of death in this subgroup, highlighting biliary events as a clinically important outcome.

Biliary-event-free survival (BEFS) was analyzed based on BED10. A Kaplan–Meier analysis showed no significant difference in BEFS between patients receiving BED10 < 55 Gy and those receiving ≥ 55 Gy (*p* 0.3). On Cox proportional hazards analysis, the BED10 dose was not significantly associated with BEFS (HR: 0.98; 95% CI: 0.94–1.03; *p* 0.48).

### 3.3. Prognostic Factors and Patterns of Recurrence

On UVA, age was a significant predictor for PFS (HR 1, 95% CI: 0.9–1; *p* 0.03) but not OS (HR 0.9, 95% CI: 0.9–1.1; *p* 0.11). Pre-radiation biliary obstruction was associated with worse OS (HR 2.47, 95% CI: 1.2–5.1; *p* 0.01), while elevated CA 19-9 levels at baseline were associated with worse PFS (HR 1, 95% CI: 1–1; *p* 0.04), as was lesion size (in mm) (HR 1.01, 95% CI: 1.01–1.03; *p* 0.004). Interestingly, the RT dose (BED10) or prior chemotherapy were not associated with OS or PFS. On MVA, only lesion size (in mm) was statistically significantly associated with worse PFS (HR 1.6, 95% CI: 1.2–2.2; *p* 0.0048). The findings are tabulated in Table 3.

**Figure 4:**
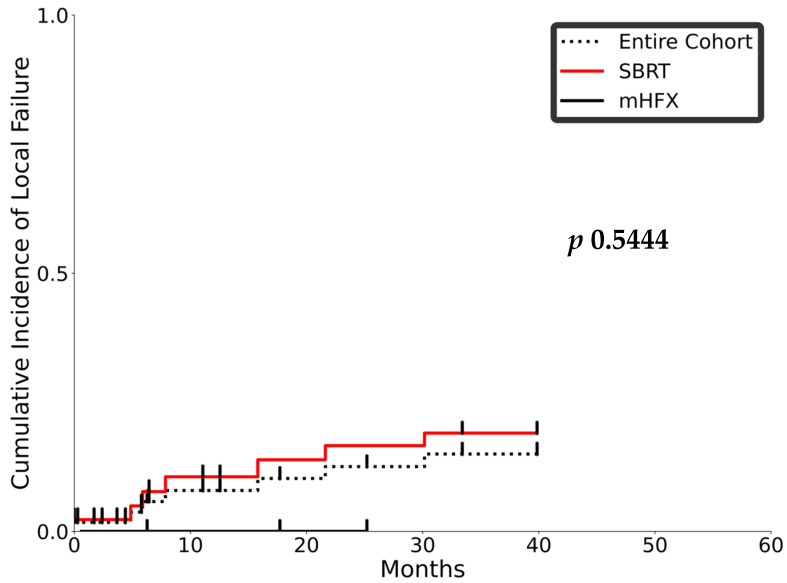
Cumulative incidence function (CIF) plot for local recurrence (LR) over time. The black dotted, red solid, and black solid lines represent the medians for the entire cohort, the subgroup treated with SBRT, and the subgroup treated with mHFX, respectively. The ticks denote censoring.

Overall, disease progression was documented in 37 patients (66%). The most common site of disease progression was intrahepatic. Most intrahepatic recurrences were due to new liver lesions (*n* = 24; 64.9%). Only five patients (13.5%) had isolated LR. The LR rates were 7.9% at one year and 12.5% at one and two years, and, as noted in Figure 4, there was no statistically significant difference between the SBRT and moderately hypofractionated RT. The intrahepatic recurrence rates at one and two years were 26% and 29.3%, respectively; the same was 31.2% and 33.4% in the SBRT cohort and 6.9% and 13.1% in the mHFX cohort. Interestingly, only five (20.8%) of the intrahepatic recurrences were in the high-dose region, while three (12.5%) were in the elective region. More commonly, (*n* = 14; 58.3%) recurrences were noted both in and out of the field. There was no significant difference in local control with doses (BED10) less or more than 80.5 Gy (*p* 0.2).

**Figure 5:**
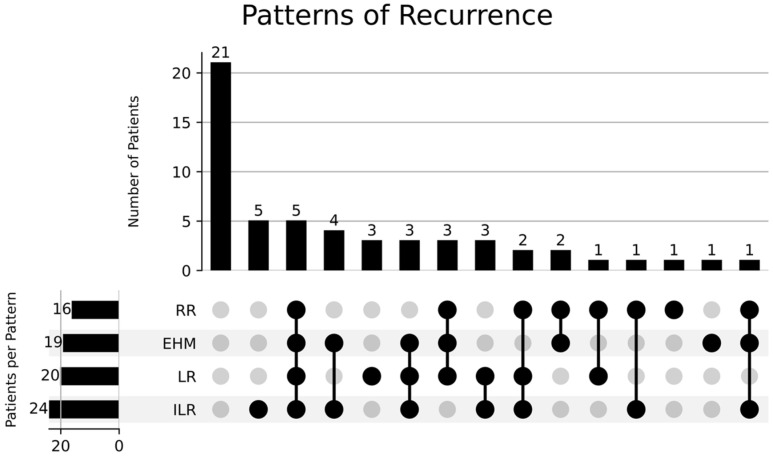
UpSet plot illustrating recurrence patterns in patients with inoperable cholangiocarcinoma treated with radiotherapy. Each vertical bar represents the number of patients experiencing a specific combination of recurrence types, indicated by the filled circles below the bar. The horizontal bars show the total number of patients with each individual recurrence type: local recurrence (LR), regional recurrence (RR), extrahepatic metastasis (EHM), and new liver lesions (ILR).

The RR rates at one and two years were 22.9% and 28%, 27.3% and 31.6%, 6.9% and 12.1% in the entire cohort, SBRT cohort, and moderately hypofractionated cohorts, respectively. Similarly, patients with isolated EHM were few (*n* = 6; 16.2%). Only a few cases of isolated RR were noted (*n* = 2, 5.4%). The rates of DM were 26% at one year and 29.3% at two years. On subgroup analysis, the patients treated with SBRT had DM rates of 31.3% at one year and 33.4% at two years. For the moderately hypofractionated cohort, they were 6.8% and 12.6% at one and two years, respectively. Lastly, five patients (13.5%) had disease progression at all sites (intrahepatic, regional, and extrahepatic). The patterns of recurrence are depicted in Figure 5.

## 4. Discussion

CCA includes a range of biliary malignancies that may arise from any part of the biliary tract. Systemic therapy has been the backbone of treatment for unresectable disease. With a high propensity for distant metastases, the role of local treatment in unresectable CCA is not well defined. However, given the intrahepatic progression and the resultant worsening hepatobiliary function contributing to mortality and morbidity in this group of patients, local therapy can potentially delay the onset of liver failure and improve quality of life and even survival [9]. Murakami et al. reported a two-year overall survival (OS) of 35.7% with conventionally fractionated external beam radiotherapy (median dose: 54 Gy in 1.8–3 Gy per fraction), though durable local control beyond two years was achieved in only 19% of patients [10]. In comparison, Jung et al. demonstrated substantially higher local control rates with ultra-hypofractionated SBRT (45 Gy in three fractions), reporting one-year and two-year LC of 85% and 72%, respectively [11]. A small prospective study by Polistina et al. combining SBRT (30 Gy in three fractions) with concurrent gemcitabine reported a two-year OS of 80%, though this declined to 30% by year four, suggesting that initial gains may not translate into durable survival benefits [12]. Conversely, Kopek et al. observed a median OS of just 10.6 months with SBRT in a similar population, underscoring the variability in outcomes across studies [13]. In a retrospective review of 66 patients with inoperable iCCA treated with hypofractionated photon or proton RT (median dose 58.05 Gy in 15 fractions (range 37.5–67.5 Gy)), Smart et al. reported a two-year OS of 58% and LC of 84% with grade three toxicities of about 11% [14]. A phase II multi-institutional study reported a median OS of 21 months (95% CI: 13–29), and 96% LC at two years [15] with hypofractionated proton beam therapy. The median RT dose was 58 Gy (range 15.1–67.5); 14% (*n* = 6) developed grade three side effects. The current NCCN guideline recommendations include definitive RT with/without systemic therapy as a treatment option. There is a strong recommendation [16] for considering RT as a definitive or consolidative treatment in unresectable iCCA following systemic therapy. In this retrospective analysis of 56 patients with cholangiocarcinoma (CCA) treated with radiotherapy (RT), the median overall survival (OS) was 20 months, and the median progression-free survival (PFS) was 10 months, with two-year OS and PFS rates of 29.6% and 14.9%, respectively. The LC of irradiated tumors were 92.1% at one year and 87.5% at two years. Most recurrences were intrahepatic (66%), predominantly manifesting as new liver lesions (64.9%), with only a small proportion (20.8%) in high-dose regions. Prognostic factors for worse outcomes included pre-radiation biliary obstruction, elevated baseline CA 19-9 levels, and larger lesion sizes. Radiation dose (BED10) and prior chemotherapy were not associated with OS or PFS.

The role of SBRT for CCA has been reported by other groups. The results of the present study noted an OS of 20 months using hypofractionated RT, with similar outcomes between the SBRT and moderately hypofractionated groups. Our results are comparable with prior reports (Table 4) which suggest two-year OS rates of 19–37%, except for notable outliers like the 61% reported by Tao et al. [17]. Local control/recurrence rates vary considerably across studies, from 32.7 to 79%. Mahadevan et al. reported a local control rate of 79% [18]. The local recurrence rate in our cohort was 12.5% at two years. It is worth noting that the median tumor size tended to be larger (63 mm, range 8–139 mm) than most of the reported studies. Predominant progression patterns also differ between studies, where reported. For instance, Tao et al. [17] and Brunner et al. [19] have found the predominant failure to be local, with Tao et al. suggesting that most failures fall within the high-dose region. Conversely, our present study, as well as Gkika et al. [20] and Barney et al. [21], suggests out-of-field intrahepatic failure to be most prominent. The definition of local control (treated lesion vs. any liver lesion failure) may have contributed to some of the discrepancies in these results. Interestingly, our study did not find a significant correlation between radiotherapy dose (BED10) and PFS or OS, unlike some other studies. A dose–response analysis from M.D Anderson Cancer Center [17] noted BED to be the most important prognostic factor for OS, with outcomes comparable to surgical resection when BED was > 80.5 Gy (three-year OS 73% vs. 38%, *p* 0.017, three-year LC 78% vs. 45%, *p* 0.04). Brunner et al. [19] reported a median survival of 24 months with BED at doses > 91 Gy versus lower doses and a LC of 91% at 12 months with higher doses.

The role of nodal irradiation is another question that requires clarification. Kozak et al. reported an all-site regional recurrence/progression rate of 13%, 24% in hCCAs, at one year with SBRT (median dose 40 Gy; median number of fractions 5) [22]. The authors concluded that elective nodal irradiation should be considered with SBRT. However, given the poor overall survival and high distant metastasis rate, the benefits of regional irradiation remain uncertain. For patients who are at a reasonably low risk of toxicity, regional nodal irradiation remains a consideration for clinicians. In the present study, we report high regional control rates (two-year RC of 72%). Our study suggests that these patients are prone to hospitalization and biliary toxicity. However, given the high rates of disease progression, the attribution of the stated adverse events to treatment versus disease progression remains unclear and difficult to discern. Clinicians must be reminded of the adverse event rate in making clinical decisions. An overview of the outcomes and patterns of recurrence in the studies mentioned above, along with the present study, is recorded in Table 4.

**Table 4:** Studies of CCA treated with SBRT and mHFX. * Patients received concurrent gemcitabine at 1000 mg/m^2^ weekly during SBRT and after completion. N/A: not available.

Author, Year	Nature of Study	Disease Type	No. of Patients	Median Tumor Size (mm) (Range)	Median GTV Volume (cc) (Range)	Median PTV Volume (cc) (Range)	Radiation Dose Fractionation	Local Control (LC) at 2 Years	OS and PFS at 2 Years	Predominant Pattern of Progression
Murakami et al. [10]	Retrospective	Histologically or radiologically proven iCCA 7 (33.3%) dCCA 11 (52.4%) Border 3 (14.3%)	21	N/A	N/A	N/A	54 Gy (45–60 Gy at 1.8–3 Gy per fraction); BED 63.7 Gy (53.1–78 Gy)	32.7%	OS 35.7% PFS 16.1%	No prophylactic nodal RT
Jung et al. [11]	Retrospective	Histologically or radiologically proven iCCA 33 (57%) dCCA 25 (43%)	58	N/A	40 (5–1287)	N/A	45 Gy in 3 fractions (15–60 Gy in 1–5 fractions); BED 86 Gy (48–150 Gy)	72%	OS 20%	Not reported
Polistina et al. * [12]	Prospective	hCCA	10	N/A	N/A	N/A	30 Gy in 3 fractions	N/A	OS 80%	Local progression (*n* = 6, 60%), intra- and extrahepatic (*n* = 6, 60%)
Kopek et al. [13]	Retrospective	iCCA 1 (4%) pCCA 26 (96%)	27	N/A	N/A	N/A	45 Gy in 3 fractions BED10 Gy 112.5	N/A	N/A	Out-of-field liver (*n* = 5)
Tao et al. [17]	Retrospective	Histologically proven inoperable iCCA	79	79 (22–170)	198 (12–966)	548 (55–2.012)	58.05 Gy (35–100 Gy in 3 to 30 fractions); BED 80.5 Gy (43.75–180 Gy)	45%	OS 61% PFS 61%	High-dose region (*n* = 34; 89%)
Mahadevan et al. [18]	Retrospective	iCCA 31 (73.8%) iCCA + dCCA 9 (21.4%) hCCA 2 (4.8%)	34	N/A	63.8 cc (5.88–500.56)	N/A	30 Gy (10–45) in 3 (1–5) fractions BED10 Gy 60 (20–85.5)	N/A	OS 31%	Distant metastasis (*n* = 9, 69.2%)
Brunner et al. [19]	Retrospective	iCCA 41 (50%) hCCA 31 (38%) dCCA 3 (4%) N/A 7 (9%)	64	44 (10–180)	N/A	114 (5–1876)	BED 67.2 Gy (36–115 Gy) in 3–17 fractions	73%	OS 34%	Local (*n* = 14, 18%)
Gkika et al. [20]	Retrospective	iCCA 17 (40%) dCCA 26 (60%)	37	49 (20–180)	N/A	124 (9–1356)	45 Gy (25–66 Gy in 3–12 fractions); BED 67.2 Gy (30–102 Gy)	58%	OS 25% PFS 19%	Out-of-field liver failure (*n* = 21, 72.4%)
Barney et al. [21]	Retrospective	iCCA 6 (60%) hCCA 3 (30%) Adrenal met 1 (10%)	10	N/A	N/A	79.1 cc (16.0–412.4)	55 Gy (45–60) in 5 (3–5) fractions BED10 Gy 115.5 (112.5–132)	N/A	N/A	Out-of-field liver (*n* = 5, 50%)
Momm et al. [23]	Retrospective	dCCA	13	N/A	N/A	190.2 (47–393)	32–56 Gy, 3–4 Gy/week BED10 Gy	N/A	N/A	
Tse et al. [24]	Phase 1	iCCA	41 (iCCA *n* = 10)	N/A	172 (10–465)	N/A	32.5 Gy (28.2–48.0 Gy) in 6 fractions BED 50.1 Gy (41.5–104.4 Gy)	65% at 1 year	OS 58% (95% CI, 23–82%) at 1 year	Out-of-field failure
Present study	Retrospective	iCCA 44 (78.6%) dCCA 8 (14.3%) hCCA 4 (7.1%)	56	63 (8–139)		335 (14.3–3044.2)	36 (25–58.05) in 6 (5–20) fractions BED10 Gy 55 (37.5–102.6)	2-year LF 12.5%	OS 29.6% PFS 14.9%	

In this study, patients who developed post-radiotherapy biliary complications had markedly inferior overall survival rates compared to those without such events (median OS: 10.3 vs. 19.1 months), with a nearly fourfold increase in the hazard of death. While gastrointestinal toxicity remains the most frequently reported adverse event in liver-directed radiotherapy, biliary complications, as causes of treatment disruption, morbidity, and mortality, remain undercharacterized. These events are often multifactorial—arising from underlying disease burden and progression, prior biliary interventions, and potential radiation-induced injury. Collectively, they can precipitate serious clinical deterioration, including cholangitis, hepatic decompensation, or delays in systemic therapy. Kopek et al. [13] noted biliary events following SBRT in patients with cholangiocarcinoma, and Momm et al. [23] previously reported cholangitis as the cause of the early discontinuation of external beam radiotherapy. Tse et al. [24] reported five percent (*n* = two) of patients developing transient biliary obstruction after a few fractions and a worsening of liver function from Child–Pugh class A to B within three months post-RT. Despite these observations, biliary toxicity is often underreported or subsumed under general gastrointestinal adverse events, obscuring its prognostic relevance. Nonetheless, our data argues that in cases of biliary event risk due to disease, clinicians should consider radiotherapy to potentially avert such outcomes, while utilizing radiation doses that are less likely to lead to injury. Nevertheless, their strong association with overall survival underscores their clinical relevance, regardless of causality. To our knowledge, this is the first study to quantitatively demonstrate an association between post-treatment biliary complications and overall survival in patients with inoperable cholangiocarcinoma treated with moderately or ultra-hypofractionated radiotherapy. The median survival in those who developed biliary complications was significantly lower than those without biliary complications (10.4 months vs. 19.1 months, *p* 0.0009). Among the patients without biliary complications, 78% survived beyond 12 months and 58% beyond 18 months, indicating that a meaningful subset can achieve durable outcomes following radiotherapy. The observed survival gap highlights the need to consider biliary-event-free survival (BEFS) as a distinct and clinically relevant endpoint in future prospective trials, particularly those evaluating SBRT or other liver-directed therapies.

There are limitations to our study. There have been significant changes in the landscape of systemic therapy in CCAs and combination treatments. For example, the results from a phase 2 randomized ABC-07 trial did not meet the primary endpoint of PFS by adding SBRT to gemcitabine and cisplatin but noted better primary tumor control [25]. On the other hand, a phase 2 single-arm study showed a median PFS of 12 months with the addition of SBRT to the anti-PD1 drug camrelizumab [26]. A formal evaluation of a combination of systemic and local RT, though warranted, could not be conducted from this patient cohort spanning almost two decades. As with many retrospective studies, a definitive attribution of biliary events to radiotherapy versus disease progression was not possible. A further shortcoming in the study lies in the fact that all of these cholangiocarcinomas (intrahepatic, extrahepatic, and hilar) were grouped together, though patients eligible for standard therapies would be treated differently. Future studies seeking to test the role of the addition of hypofractionated RT to standard therapies should be mindful of the different challenges in treating these complex diseases.

## 5. Conclusions

This retrospective study evaluated outcomes in patients with inoperable cholangiocarcinoma treated with moderately or ultra-hypofractionated radiotherapy at a tertiary academic center over an extended period of time. Intrahepatic disease progression—particularly in untreated liver segments—was common, underscoring the need for improved strategies to enhance intrahepatic control. No significant differences in survival were observed between SBRT and moderately hypofractionated regimens, suggesting that both may be appropriate when tailored to a patient’s condition and disease burden. Notably, this study provides the first quantitative evidence linking post-radiotherapy biliary complications with significantly inferior overall survival—an association that has received limited attention in the prior literature. Given that biliary events may result from both treatment-related toxicity and disease progression, their impact on survival reinforces the importance of careful treatment planning and vigilant post-treatment monitoring. On Cox proportional hazards analysis, BED was not significantly associated with BEFS, suggesting that factors beyond dose intensity may contribute to the risk of biliary complications. While retrospective in nature and limited by disease heterogeneity and incomplete toxicity grading, the study is distinguished by its detailed characterization of radiotherapy parameters, in-depth recurrence mapping, and the novel quantitative assessment of biliary complications as a prognostic factor. These findings support the inclusion of BEFS as a meaningful endpoint in future prospective trials. As systemic therapy options continue to evolve, better patient selection for radiotherapy and innovative local treatment approaches will be essential—not only for improving distant control, but also for reducing morbidity and enhancing survival in this challenging malignancy.

## Figures and Tables

**Table 1:** Baseline patient and disease characteristics and pre-radiotherapy treatment.

**Baseline Patient Characteristics**	
Age (mean, range)	67.5 years (38–90)
Female (*n*, %)	24 (42.9%)
Male (*n*, %)	32 (57.1%)
Presence of Cirrhosis (*n*, %)	8 (14.3%)
**Baseline** **Tumor Characteristics**	
iCCA (*n*, %)	44 (78.6%)
dCCA (*n*, %)	8 (14.3%)
hCCA (*n*, %)	4 (7.1%)
Lesion Size (mm) (median, range)	63 (8–139)
Presence of Satellite Nodules (*n*, %)	12 (21.4%)
Absence of Satellite Nodules (*n*, %)	44 (78.6%)
Node-Positive (*n*, %)	21 (37.5%)
Node-Negative (*n*, %)	35 (62.5%)
**Baseline CA 19-9** (U/mL) (median, range)	2185 (0–37,113)
**Treatment**	
**PreRT** **Biliary Intervention**	
No (*n*,%)	39 (69.6%)
Yes (*n*,%)	17 (30.4%)
**Type of Biliary Interventions**	
Stent (*n*,%)	8 (7.8%)
Percutaneous Drain (*n*,%)	6 (14.3%)
Both (*n*,%)	3 (5.4%)
**Upfront** **Systemic Therapy**	
No (*n*,%)	33 (58.9%)
Yes (*n*,%)	23 (41.1%)
**Systemic Therapy Agents Used**	
Gemcitabine (*n*, %)	1 (1.8%)
Gemcitabine + Capecitabine (*n*, %)	2 (3.6%)
Cisplatin + Gemcitabine (*n*, %)	18 (32.1%)
Cisplatin + Gemcitabine + Durvalumab (*n*, %)	2 (3.6%)
**Cycles of Systemic Therapy Received** (median, range)	7 (4–47)
**Post-RT CA 19-9** (U/mL) (median, range)	67 (16–10,000)

CA 19-9, cancer antigen 19-9. Cycles of systemic therapy received reflect the number of cycles received by patients who received any systemic therapy.

**Table 2:** Radiation treatment parameters.

Radiotherapy Details	Entire Cohort (*n* = 56)	SBRT (*n* = 43)	mHFX (*n* = 13)
Dose prescribed (median, range)	36 Gy (25–58.05)	32 Gy (25–54)	52.5 Gy (36–58.05)
BED_10_ (median, range)	55 Gy (37.5–102.6)	51.2 Gy (37.5–102.6)	70.9 Gy (46.8–80.5)
Fractions prescribed (median, range)	6 (5–20)	6 (5–6)	15 (12–20)
Overall treatment time (median, range)	13 days (1–24)	12 days (10–24)	21 days (16–22)
High-dose CTV volume (median, range)	152.5 cc (5.7–2320.2)	165.6 cc (5.7–2320.2)	138.9 cc (39.7–309)
PTV volume (median, range)	335 cc (14.3–3044.2)	362.3 cc (14.3–3044.2)	331 cc (69.3–677.4)
Mean liver dose (median, range)	15 Gy (1.3–23.7)	13.7 Gy (1.3–21)	22.4 Gy (9.2–23.7)
D0.5 cc to small bowel (median, range)	11.4 Gy (0.1–39.7)	10.3 Gy (0.1–33.4)	12.3 Gy (2.5–39.7)
D0.5 cc to duodenum (median, range)	25.4 Gy (0.5–41.2)	17 Gy (0.5–33.7)	38.3 Gy (4.5–41.2)
D0.5 cc to stomach (median, range)	23.9 Gy (0.9–40)	21 Gy (0.9–35.3)	38.1 Gy (20.5–40)

Gy: Gray; Cc: cubic centimeters; BED: biologically effective dose; CTV: clinical target volume; PTV: planning target volume; D0.5 cc: minimum dose to 0.5 cc receiving highest dose. BED calculation was performed with an α/β assumption of 10 for the tumor dose. Mean liver dose is calculated as mean dose received by the volume of the liver with the tumor subtracted.

**Table 3:** Univariate analyses for OS and PFS; *p*-values and hazard ratios with 95% confidence intervals.

Factors	OS		PFS	
	HR (95% CI)	*p* Value	HR (95% CI)	*p* Value
Age (years)	0.9 (0.9–1.1)	0.11	0.97 (0.94–0.99)	0.03
Baseline CA 19-9 (U/mL)	1 (0.99–1)	0.19	1 (1–1)	0.04
Types (iCCA/hCCA/dCCA)	1.29 (0.78–2.11)	0.32	1.48 (0.92–2.39)	0.11
Lesion size (mm)	1 (0.99–1.01)	0.48	1.01 (1–1.02)	0.004
Pre-radiation biliary obstruction (present/absent)	2.47 (1.2–5.1)	0.01	0.83 (0.36–1.91)	0.66
Pre-radiation chemotherapy (present/absent)	1.86 (0.91–3.78)	0.09	1.79 (0.91–3.53)	0.11
PTV volume (cc)	1 (0.99–1)	0.37	1 (0.99–1)	0.06
RT dose (BED10 Gy)	0.99 (0.97–1)	0.54	0.99 (0.97–1.01)	0.57
Overall treatment time (days)	0.98 (0.91–1.05)	0.03	0.97 (0.94–0.99)	0.9
Post-RT CA 19-9 (U/mL)	1 (0.99–1)	0.22	1.1 (0.9–1.4)	0.24

## Data Availability

Research data are not available at this time.
